# Combined Use of Diagnostic Fumarate Addition Metabolites and Genes Provides Evidence for Anaerobic Hydrocarbon Biodegradation in Contaminated Groundwater

**DOI:** 10.3390/microorganisms8101532

**Published:** 2020-10-06

**Authors:** Gurpreet Kharey, Gabrielle Scheffer, Lisa M. Gieg

**Affiliations:** Department of Biological Sciences, University of Calgary, 2500 University Drive NW, Calgary, AB T2N 1N4, Canada; gurpreet.kharey@mail.mcgill.ca (G.K.); gabrielle.scheffe1@ucalgary.ca (G.S.)

**Keywords:** hydrocarbon-contaminated groundwater, anaerobic biodegradation, hydrocarbon bioremediation, fumarate addition metabolites, *assA* gene, *bssA* gene

## Abstract

The widespread use of hydrocarbon-based fuels has led to the contamination of many natural environments due to accidental spills or leaks. While anaerobic microorganisms indigenous to many fuel-contaminated groundwater sites can play a role in site remediation (e.g., monitored natural attenuation, MNA) via hydrocarbon biodegradation, multiple lines of evidence in support of such bioremediation are required. In this study, we investigated two fuel-contaminated groundwater sites for their potential to be managed by MNA. Microbial community composition, biogeochemical indicators, fumarate addition metabolites, and genes diagnostic of both alkane and alkyl-monoaromatic hydrocarbon activation were assessed. Fumarate addition metabolites and catabolic genes were detected for both classes of hydrocarbon biodegradation at both sites, providing strong evidence for in situ anaerobic hydrocarbon biodegradation. However, relevant metabolites and genes did not consistently co-occur within all groundwater samples. Using newly designed mixtures of quantitative polymerase chain reaction (qPCR) primers to target diverse *assA* and *bssA* genes, we measured *assA* gene abundances ranging from 10^5^–10^8^ copies/L, and *bssA* gene abundances ranging from 10^5^–10^10^ copies/L at the sites. Overall, this study demonstrates the value of investigating fuel-contaminated sites using both metabolites and genes diagnostic of anaerobic hydrocarbon biodegradation for different classes of hydrocarbons to help assess field sites for management by MNA.

## 1. Introduction

The use of petroleum-based fuels to serve our global energy needs has resulted in the contamination of many pristine environments with hydrocarbons due to failing infrastructure or accidental spills. Hydrocarbons, compounds that are composed solely of carbon and hydrogen, are the primary components of refined fuel mixtures such as gasoline and diesel. Such mixtures are often stored in metal tanks either above-ground or underground which can corrode, potentially leaking hydrocarbons into the environment. Once introduced to groundwater, hydrocarbon contaminants can be carried away from the source due to groundwater flow and potentially enter drinking water sources, other bodies of water, or housing infrastructure. Exposure to these chemicals may lead to the development of chronic illnesses, such as nasal and renal cancers, leukemia, and lower quality of life [1,2].

Research within the last 3 decades has clearly demonstrated that microorganisms inhabiting anoxic environments such as deep subsurface petroliferous reservoirs and shallow groundwater aquifers can utilize hydrocarbons as their carbon and energy sources [3,4,5]. In the absence of oxygen that serves as both a terminal electron acceptor and a powerful oxidant, anaerobic microorganisms employ distinct mechanisms for overcoming the stability of hydrocarbons for use as carbon and energy sources [6]. For unsubstituted hydrocarbons, such as benzene, naphthalene, or phenanthrene, evidence is increasingly pointing to carboxylation as an important activation step [7,8,9,10], although the mechanism is not fully understood. Alkyl-substituted monoaromatic hydrocarbons (such as toluene, ethylbenzene, and xylene, [TEX]), alkyl-substituted polycyclic aromatic compounds (PAHs), and *n*-alkanes are activated by addition to fumarate [5,6], aside from a few exceptions [11]. This mechanism, commonly referred to as ‘fumarate addition’, is the most well-studied mechanism of hydrocarbon activation under anoxic conditions, and will be the focus of the work described here.

To date, three fumarate addition enzymes (FAEs) have been characterized to varying extents. Benzylsuccinate synthase (BSS) catalyzes the addition of fumarate to toluene [12,13] and potentially, closely related alkylated monoaromatic hydrocarbons such as *o-, m-,* and *p-*xylene and ethylbenzene [14,15]. Alkylsuccinate synthase (ASS, also known as 1-methylalkylsuccinate synthase, MAS) catalyzes the addition of the subterminal carbon of an alkane to the double bond of fumarate, forming an ‘alkylsuccinate’ [16,17]. Naphthylmethylsuccinate synthase (NMS) is the presumed paralog to BSS catalyzing fumarate addition to methyl-substituted PAHs [18]. These reactions, best understood for BSS and ASS, produce initial ‘succinate’ intermediates that are further metabolized by beta-oxidation-like reactions. Genes for catalytic subunits of these enzymes, denoted as *bssA* or *assA* (*masD*), have also been identified [14,16,17,19], and are known to be highly diverse across multiple environments and clades [19,20,21,22,23,24].

Many techniques can be used to determine whether a hydrocarbon-contaminated groundwater site is undergoing in situ bioremediation [25] and it is critical to use a combined approach to provide multiple lines of evidence in support of such natural attenuation [4,26]. Since being proposed as unique indicators of anaerobic hydrocarbon biodegradation in contaminated groundwater [27], fumarate addition metabolites have been detected at many fuel-impacted field sites [5]. As these metabolites have a specific relationship to their parent hydrocarbon and are not found in fuel mixtures, their detection in contaminated groundwater provides strong evidence that the associated microbial community members are capable of in situ anaerobic hydrocarbon biodegradation. However, the transient nature of these biodegradation pathway intermediates can make their detection difficult as they rarely accumulate to higher than nanomolar concentrations in situ [5,28]. As an alternate approach, polymerase chain reaction (PCR)-based assays targeting a dedicated gene that encodes a subunit of BSS (or more rarely, ASS) have also been applied to contaminated groundwater aquifers to indicate anaerobic hydrocarbon degradation potential via fumarate addition [19,29,30,31,32]. These assays only measure the potential for fumarate addition, as the presence of or quantity of a gene (e.g., number of copies per unit volume) does not denote its actual expression or the activity of the translated protein [4]. Two previous studies have sought both fumarate addition metabolites and associated genes as biomarkers to garner evidence for anaerobic hydrocarbon biodegradation in groundwater environments, focussing on the *bssA* gene [30,32]. Oka et al. [32] detected the *bssA* gene at one well location, while metabolites were detected in multiple wells at the contaminated groundwater site under study. Using two test lanes (distinct paths of groundwater flow) to examine the effects of ethanol (a gasoline oxygenate) on benzene, toluene, and *o*-xylene biodegradation (one lane injected with ethanol and BTX, one lane injected with BTX only), Beller et al. [30] detected *bssA* genes in all groundwater wells tested. However, fumarate addition metabolites were primarily detected only in the ethanol-supplemented lane, presumably because the presence of this labile substrate led to slower rates of hydrocarbon biodegradation and higher benzylsuccinate concentrations [30]. Both of these studies showed that interrogating contaminated sites for both known biodegradation genes and metabolites can help to overcome the limitations of using one approach alone in order to provide evidence in support of in situ hydrocarbon biodegradation.

In this study, we evaluated two hydrocarbon-contaminated groundwater sites for their potential to be managed by in situ bioremediation (e.g., by monitored natural attenuation, MNA) by investigating each site using multiple approaches that included measurements of fumarate addition metabolites and FAE genes. Metabolites and genes diagnostic of both anaerobic alkane and alkyl-monoaromatic hydrocarbon biodegradation were sought. As part of this study, we also designed new quantitative polymerase chain reaction (qPCR) primer mixtures for broad capture of both *assA* and *bssA* genes, and used these to quantify these biodegradation genes in groundwater samples. As the two sites examined were distinct in their levels of hydrocarbon concentrations (comparatively low and high), the study also provided the opportunity to assess whether there were any relationships between biodegradation gene abundances and hydrocarbon concentrations.

## 2. Materials and Methods

### 2.1. Site Descriptions

**Figure 1:**
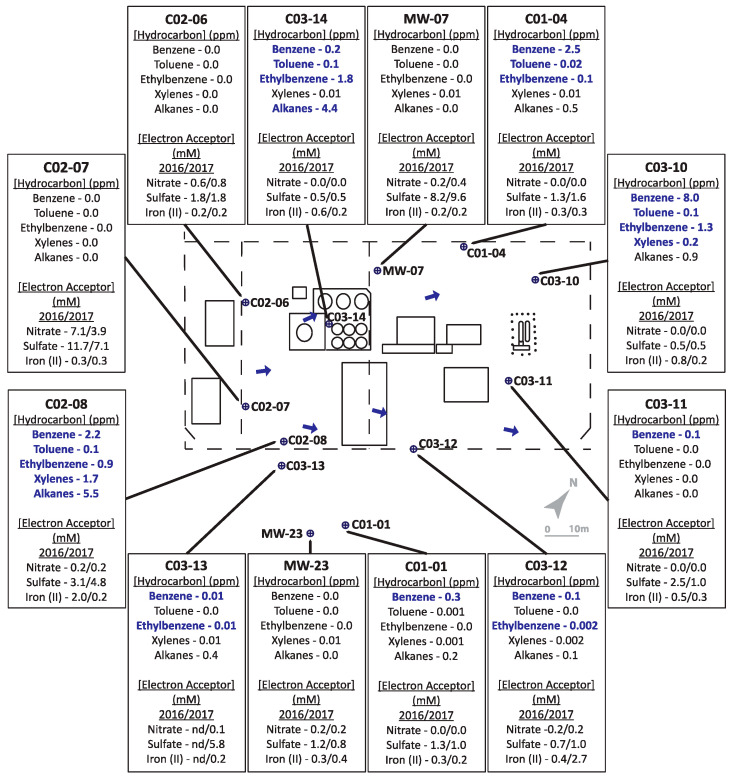
Schematic of Site A, with pop-outs summarizing hydrocarbon concentrations (analyzed in 2016 only) and indicators of potential electron accepting processes (analyzed in 2016 and 2017). Bolded text indicates hydrocarbon concentrations above allowable limits for hydrocarbons in non-drinking groundwater sources. ‘nd’ = no data available as well was not sampled.

**Figure 2:**
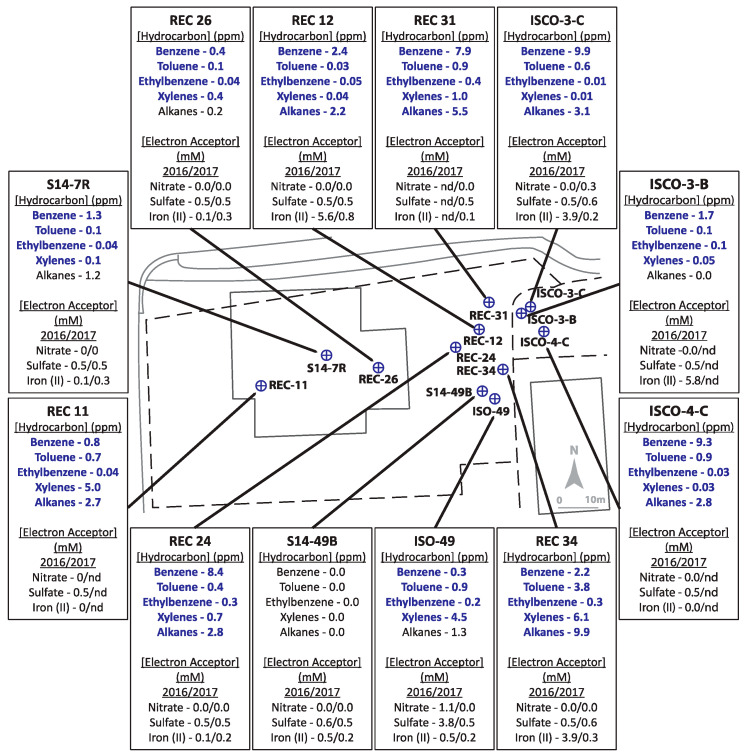
Schematic of Site B, with pop-outs summarizing hydrocarbon concentrations (analyzed in 2016 only) and indicators of potential electron accepting processes (analyzed in 2016 and 2017). Bolded text indicates hydrocarbon concentrations above allowable limits for hydrocarbons in non-drinking groundwater sources. ‘nd’ = no data available as well was not sampled.

The two hydrocarbon-contaminated sites under study (designated Site A and Site B) are located in Alberta, Canada. Both field sites are former gasoline and diesel storage and dispensing stations that leaked these fuel mixtures into their underlying shallow groundwater aquifers. For Site A (Figure 1), a leak was initially discovered in the early 1990s in an above-ground fuel storage tank. The bulk fuel facility was decommissioned in 2003. Land farming and other in situ bioremediation efforts were undertaken at least 10 years prior to this investigation yielding inconclusive clean-up results due to limited sampling. The geology of Site A is considered predominantly clayey-silt, with groundwater levels averaging ~1.4 metres below ground surface (mBGS), underlain by bedrock at ~2 mBGS. Groundwater flow is generally towards the northeast (Figure 1). Site B (Figure 2) is primarily clay to silty-clay in composition, with groundwater levels ranging between 0.5–1.7 mBGS. Hydrocarbon releases occurred from above ground and underground storage tanks at different times, resulting in two hydrocarbon plumes and complex groundwater flow patterns, though groundwater flow was generally eastward (towards structures shown on right of the Figure 2 schematic). Numerous undisclosed remediation strategies had been used at Site B prior to the initiation of this study. Both sites were selected for study by industry partners as they were potential candidates for monitored natural attenuation or other bioremediation approaches but had not been microbiologically characterized. Twelve groundwater monitoring wells, that included both non-contaminated and hydrocarbon-contaminated locations, were selected from each site for the study based on prior monitoring efforts.

### 2.2. Sample Collection

Groundwater sampling was carried out by initially removing 6 L of groundwater to purge the wells. Two 1 L volumes of water sample from each groundwater well were then taken, completely filling 1 L glass bottles to minimize air ingress. One bottle contained 5 mL 6 M HCl such that the water sample became acidified to pH 2; this bottle was used for hydrocarbon metabolite analysis. The other bottle was left unchanged, and was used for hydrocarbon biodegradation gene analysis and microbial community profiling. A ‘trip blank’, composed of tap water (municipal drinking water), was included at sampling events to account for any background hydrocarbon contamination due to sampling vessels or equipment. All sample bottles were placed on ice and transported to the University of Calgary. Upon arrival, all sample bottles were kept at 4 °C until they were processed. Samples for hydrocarbon measurements were taken at the same time but in separate collection vessels, and concentrations were measured by a third party (AGAT Laboratories, Calgary, AB; conducted in 2016 only based on limited resources). Nitrate and sulfate concentrations were measured by ion chromatography [33], while Fe(II) levels were measured using the ferrozine assay [34].

### 2.3. Hydrocarbon Metabolite Analysis

Acidified groundwater samples were extracted and processed for hydrocarbon metabolite analysis as previously described [35]. Briefly, ethyl acetate was used as the organic solvent, and concentrated extracts were silylated using *N,O*-bis(trimethylsilyl)trifluoroacetamide. These prepared samples were analyzed by gas chromatography coupled with mass spectrometry (GC/MS) as per reported methods [35]. Qualitative identification of alkylsuccinates were identified based on characteristic MS fragmentation ion profiles [5,36]. Benzylsuccinate was identified based on GC retention time and mass spectral comparison to a commercially-available standard (Alfa Aesar, Tewksbury, MA, USA), while synthesized *m*-methylbenzylsuccinate and ethylbenzylsuccinate [36] were used to identify fumarate addition metabolites from xylenes and ethylbenzene, respectively.

### 2.4. Biomass Collection and DNA Extraction

Non-acidified samples were centrifuged at 10,000 rpm for 10 min using a Beckman Coulter Avanti J-E series centrifuge unit (Beckman Coulter, Brea, CA, USA). Collected pellets were re-suspended in approximately 10 mL of supernatant and stored at −80 °C. DNA from pellet biomass was extracted using the MP Bio FastDNA Extraction Kit for Soil (MP Biomedicals, Solon, OH, USA) as per the manufacturer’s instructions. Extracted gDNA was quantified using fluorometry (Qubit 2.0 fluorometer; sample volume 1 μL). Sample gDNA concentrations were not normalized to preserve possible less abundant taxa.

### 2.5. 16S rRNA Gene Analysis for Microbial Community Profiling

Microbial community analysis was done using the same DNA preparation procedure as described above. Microbial community composition was assessed using Illumina MiSeq sequencing of the V6-V8 hypervariable region, amplified using primers Illumina926f (TCGTCGGCAGCGTCAGATGTGTATAAGAGACAGAAACTYAAAKGAATTGRCGG) and Illumina1392r (GTCTCGTGGGCTCGGAGATGTGTATAAGAGACAGACGGGCGGTGTGTRC) [37]. Amplification was undertaken using the following protocol: first-round PCR reactions contained: 12.5 µL polymerase mastermix (Fermentas Taq (ThermoFisher Scientific, Waltham, MA, USA) or KAPA Hi-Fi (Roche, Basel, Switzerland), 0.5 µL each forward and reverse primer (200 nM), 1, 2, 5, or 10 µL of DNA template, with remaining volume of PCR-grade water up to 25 μL final volume. Second-round PCR reactions contained: 25 μL polymerase mastermix (Fermentas Taq or KAPA Hi-Fi), 1 μL each of forward and reverse primer (Illumina NexteraXT Indexes (Illumina, San Diego, CA, USA); 200 nM), 10 μL of purified first-round DNA amplicon, and 13 μL of PCR-grade water for a total reaction volume of 50 μL. Thermocycling conditions for both first- and second-round amplification were as described in Toth and Gieg [35], using either Fermantas Taq polymerase or KAPA Hi-Fi polymerase. Purifications of the first- and second-round PCR amplicons were undertaken using AMPure Magnetic beads or a Qiagen PCR Purification kit (AMPure: Beckman Coulter, Brea, CA; Qiagen, Hilden, Germany). Samples were then normalized to 2 ng/µL and pooled into a sequencing library. Samples were loaded onto the Illumina MiSeq sequencing platform (2 × 300) kit at the International Microbiome Centre (University of Calgary, Calgary, Alberta, Canada). The resulting sequencing reads were assembled using PEAR 0.9.6 (50 bp overlap, 350 bp truncation) and given taxonomic rank through MetaAmp Version 2.0 using the SILVA 132 database at a species 97% similarity cutoff [38,39]. Microbial community analyses were undertaken using a 2% relative read abundance cut-off. This was done to limit the amount of less abundant reads, ultimately simplifying the analysis to the most highly abundant taxa. 16S rRNA gene sequences have been deposited in the NCBI Short Read Archive (SRA) database with assigned accession numbers SAMN 15459469 to SAMN 15459510.

### 2.6. Qualitative and Quantitative Assays for *assA* and *bssA* Genes

Assaying field samples for the presence/absence of the *assA* and *bssA* genes (i.e., qualitative detection) through PCR was done using primer sets and thermocycling conditions as previously reported [19,20,22,40]. Primer sets used to interrogate all field samples were: *assA* = 7757af/8543r [22]; *bssA* = 7772f/8546r [19]. The protocol for the assay was as follows: 12.5 µL of KAPA Hi-Fi PCR MasterMix, 10.5 µL PCR-grade H_2_O, 0.5 µL each of forward and reverse primer (200 nM), and 1 µL of gDNA template. Confirmation of amplification was undertaken using gel electrophoresis (1% agarose) by comparing PCR products with the *bssA* or *assA* gene amplified from organisms known to harbour these genes (*assA, Desulfospirillum alkenivorans* AK-01 [16]; *bssA, Thauera aromatica* [12]) as positive controls. Amplicons were Sanger sequenced, and their identities were confirmed via BLASTn.

Quantification assays targeting the *assA* and *bssA* genes were performed using newly designed qPCR primer mixtures (Table 1). Details regarding primer design and validation/comparison against published primer sets are described in the Supplementary Materials. Primer mixtures used for *assA* gene quantification contained an equimolar mixture of forward primers assOri, assMsd, assEx, and assSml, with reverse primer 8543r, with an amplicon length of 486 bp (Table 1). Likewise, *bssA* gene quantification used a forward primer mixture containing an equimolar mixture of forward primers bssOil, bssMys, bssSuf, and bssWin, with reverse primer bssHitr, resulting in an amplicon length of 141 bp (Table 1). The qPCR protocol was as follows: 12.5 µL of KAPA Hi-Fi PCR MasterMix, 10.5 µL PCR-grade H_2_O, 0.5 µL each of forward and reverse primer mixtures (200 nM), and 1 µL of gDNA template. Quantification was conducted using a Bio-Rad CFX96 thermocycler, with analysis using the Bio-Rad CFXManager software, running a single threshold Cq evaluation. Thermocycling conditions for the *assA* qPCR assay were as follows: 95 °C for 3 min, 39 cycles of 95 °C for 15 s, 62 °C for 25 s (each cycle ending with a plate read), 62 °C for 2 min, followed by a melt curve (65 °C to 95 °C, increasing in 0.5 °C increments, held for 5 s, then plate read). The annealing temperature for *bssA* was adjusted to 65 °C otherwise following the same method. Amplicons used for generating standard curves were amplified using the 7772f/8546r primers [19] and gel extracted (Qiagen Gel Extraction Kit, Qiagen, Hilden, Germany) in preparation for use in a 7 log standard curve (1:10 serial dilution). Amplicon sequences were confirmed via Sanger sequencing. All qPCR amplicons were verified using the same gel electrophoresis method as described above for qualitative *assA* and *bssA* analysis, but instead using the *assA* and *bssA* qPCR primers. Those samples that did not have the appropriate length DNA bands were not included in subsequent analyses.

### 2.7. Illumina Sequencing of *assA* and *bssA* Amplicons

Those samples which matched the amplicon lengths of the standard curve amplicons using the newly designed primer mixtures were gel extracted (Qiagen Gel Extraction Kit). Primers containing the Illumina MiSeq adapter sequences (for addition of sequencing barcodes) were taken from 16S rRNA gene primers commonly used in our studies (926f/1392r) [37] and added to the 5′ end of each primer constituting the primer mix for both *assA* and *bssA* genes. MiSeq primer mix was prepared to the same concentration as the qPCR primer mix (20 μM). Thermocycling conditions were as described above for 16S amplification. Second-round PCR amplicons were gel purified the same way as in the first round. Samples were then normalized and pooled into a 2 ng/μL library. *assA* amplicons were submitted to the International Microbiome Centre (University of Calgary) for Illumina sequencing (via 2 × 300 bp sequencing kit). *bssA* amplicon sequencing was done at the University of Calgary Cumming School of Medicine Centre for Genomics and Informatics, using an Illumina MiSeq 150 × 2 kit.

Resulting sequences were assembled using PEAR 0.9.6 (50 bp overlap, 350 bp truncation) and analyzed through MetaAmp using the ‘non-16S’ option (which does not assign taxonomy) at 97% similarity [38,39]. Resulting sequences were analyzed through BLASTn [41]. Sequences matching *assA* annotated sequences or those in whole genomes of putative alkane-degrading microorganisms were selected, disregarding any sequence that returned matches with <70% coverage and identity. Sequences that passed this quality control step and thus selected for analysis were 6 of 86 for *assA* and 13 of 65 for *bssA*. Examples of remaining sequences that were neither present in the genomes of putative alkane-degrading microorganisms, nor annotated as alkylsuccinate synthase, are shown in Table S4. Selected sequences were then compiled into a Multiple Sequence Alignment (MSA) in UGENE software and aligned using ClustalW to determine similarity to other sequenced reads [42]. A maximum likelihood dendrogram was built using approximate likelihood ratio test (aLRT) tree branch support (to estimate correct placement of internal branches) and a combination of nearest neighbor interchanges (NNI) and sub-tree pruning and regrafting (SPR) tree building methods (for initial tree building when choosing which possible tree is the most likely result from given nucleotide data) [43,44]. *assA* and *bssA* amplicon sequences have been deposited in GenBank, with accession numbers MT722950 to MT722968.

## 3. Results

### 3.1. Hydrocarbons, Putative Electron Acceptors, and Microbial Community Profiles

Several sampled wells collected from both sites A (Figure 1) and B (Figure 2) were characterized by BTEX (benzene, toluene, ethylbenzene, and xylenes (all isomers)) and alkane concentrations above the Alberta guidelines for allowable concentrations in non-drinking groundwater (benzene = 0.005 ppm (mg/L); toluene = 0.024 ppm; ethylbenzene = 0.0016 ppm; xylenes (all isomers) = 0.02 ppm; alkanes = 2.2 ppm; [45]; see Table S1 in Supplementary Materials for tabulated hydrocarbon concentrations measured at both sites). Overall, Site A (Figure 1) groundwater samples were characterized by substantially lower hydrocarbon concentrations than Site B (Figure 2).

**Figure 3:**
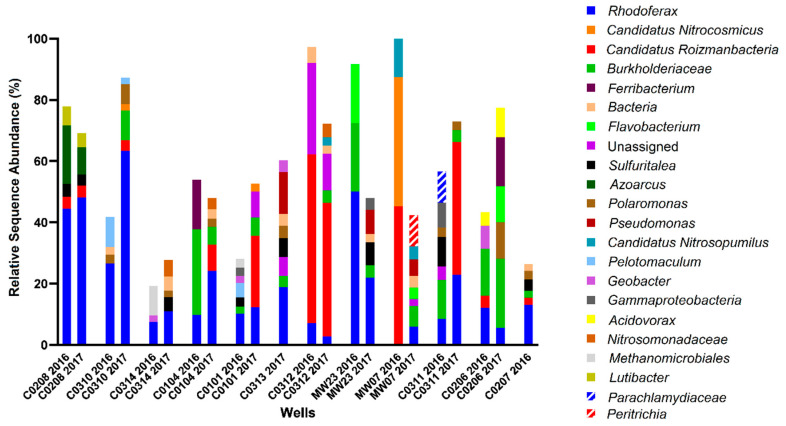
Microbial community profiles found with Site A groundwater wells in 2016 and 2017, with wells arranged in order of decreasing total hydrocarbon concentration, from left to right. Analysis was performed using MetaAmp 2.0 and the SILVA 132 database, with the 2% most abundant taxa shown to the lowest taxonomic level identified.

For Site A, hydrocarbon concentrations measured in the monitored wells in 2016 ranged between 0–8 ppm. Four of the 12 wells, located on the periphery of the site (MW-07, C02-06, C02-07, MW-23) were considered non-contaminated, as no hydrocarbons were detected above analytical limits. Well C03-10 contained the highest total BTEX concentration, while Wells C02-08 and C03-14 had the highest alkane concentrations. The remaining contaminated sampled wells contained comparatively lower, but detectable, hydrocarbon concentrations. In terms of the potential electron acceptors that were measured, one uncontaminated well contained substantial levels of nitrate (4–7 mM; well C02-07) while nitrate was comparatively low in all other contaminated wells and low or non-contaminated wells. Sulfate concentrations ranged from 5–12 mM in two of the non-contaminated wells (C02-07 and MW-07) but were lower (1–2 mM) in other non-contaminated wells (C02-06 and MW-23), suggesting that sulfate could serve as a potential electron acceptor in some locations at the site. Fe(II), the product of microbial Fe(III) reduction, was measured at less than 1 mM in most sampled wells except at two locations: C02-08 with comparatively high hydrocarbon concentrations, and C03-12 with comparatively low hydrocarbon concentrations. While the biogeochemical measurements suggested that nitrate, sulfate, or iron reduction could be electron acceptors in different portions of the site, it was difficult to discern clear trends or patterns that relate hydrocarbon concentrations with anaerobic electron accepting processes across the site. This heterogeneity was further exemplified by the microbial community profiling results, where microbial community compositions were different in each well sampled (Figure 3). While some groundwater samples showed similar taxonomic profiles in 2016 and 2017 (such as C02-08 and C03-12), most had very different taxonomic profiles across the two years. The most consistently detected taxon in all samples collected from Site A in 2016 and 2017 was *Rhodoferax*, present in all wells in the range of 3–44% (in 2016) and 5–62% (in 2017) relative sequence abundance. Members of this genus are known to have nitrate- and iron-reducing abilities, aligning with some of the terminal electron acceptor data. Many of the taxa present in some of the contaminated well samples affiliate with known hydrocarbon degraders, such as *Azoarcus*, *Geobacter*, *Polaromonas*, and some members of the *Burkholderiaceae* and *Rhodocyclaceae* families. Although sulfate was present as a putative electron acceptor in some groundwater samples, sulfate-reducing microorganisms were not prevalent at >2% relative abundance.

**Figure 4:**
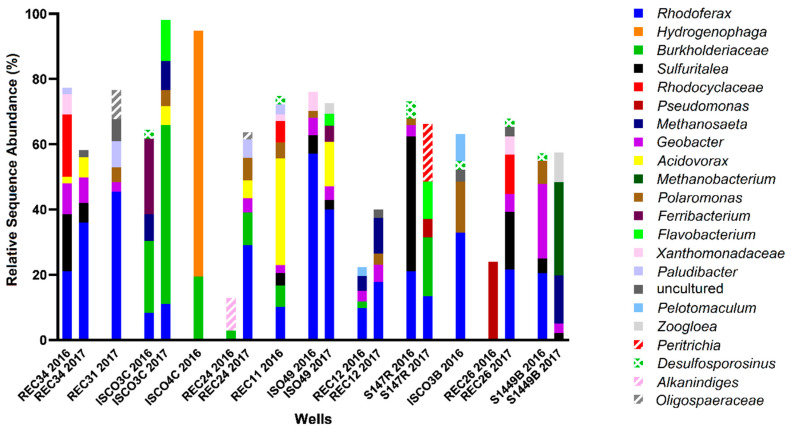
Microbial community profiles found with Site B groundwater wells in 2016 and 2017, with wells arranged in order of decreasing total hydrocarbon concentration, from left to right. Analysis was performed using MetaAmp 2.0 and the SILVA 132 database, with the 2% most abundant taxa shown to the lowest taxonomic level identified.

Site B was more highly contaminated site than Site A, with maximum total hydrocarbon concentrations reaching ~35 ppm (REC 34) with 7 wells above 10 ppm (ISCO-3-C, ISCO-4-C, ISO49, REC 11, REC 24, REC 31, and REC 34) (Figure 2, Table S1). Of the 12 wells sampled at Site B, only well S14-49B was non-contaminated, with all measured hydrocarbons below detection levels. The groundwater in all other wells surpassed the 0.005 ppm benzene guideline, the 0.024 ppm toluene guideline, the 0.0016 ppm ethylbenzene guideline, and the 0.02 ppm xylenes guideline (except for well ISCO-3-C at 0.01 ppm xylenes). In 2016, all wells except ISCO-3-B and REC 26 were above the 2.2 ppm alkane guideline. Nitrate and sulfate concentrations for almost all samples collected from in Site B were at or below 0.5 mM, aside from one well (ISO-49 in 2016). Four contaminated wells (REC12, ISCO-3-C, ISCO-3-B, and REC34) had elevated Fe(II) concentrations (4–6 mM), suggesting that iron-reducing microorganisms may be present at the site. The single non-contaminated groundwater well (S14-49B) did not show substantial concentrations of measured electron acceptors. The microbial community profiles of Site B samples collected in 2016 and 2017 are shown in Figure 4. Similar to Site A, *Rhodoferax* was present in most groundwater samples analyzed, and the microbial community profiles were different in each well sampled (Figure 4). Several taxa detected in the contaminated wells are known to have members capable of hydrocarbon biodegradation, such as *Burkholderiaceae*, *Xanthomonadaceae, Polaromonas, Geobacter, Acidovorax*, and *Desulfosporosinus*. Distinct from Site A, some methanogens were detected in Site B samples, including *Methanosaeta* and *Methanobacterium*; these taxa were most abundant in the single non-contaminated groundwater sample at Site B, S14-49B, aligning with the low levels of other potential electron acceptors (Figure 2 and Figure 4). As with Site A, no clear trends in hydrocarbon concentrations, potential electron acceptors, and taxonomic profiles could be discerned for Site B based on these measured parameters.

### 3.2. Fumarate Addition Metabolites and Biodegradation Genes

While microbial community profiling results and biogeochemical measurements revealed the presence of some putative hydrocarbon degraders and anaerobic electron acceptors at Sites A and B, heterogeneity at these sites precluded definitive evidence supporting in situ anaerobic hydrocarbon biodegradation. Therefore, additional evidence was sought based on diagnostic fumarate metabolites and genes across each site.

#### 3.2.1. Alkylsuccinates and *assA* Genes

Table 2 overviews the detection of alkylsuccinates and the *assA* gene at sampled locations across both Sites A and B. Measured alkane concentrations in each well are also indicated on Table 2. Alkylsuccinates that originated from the anaerobic biodegradation of C_5_ to C_9_ alkanes were detected at Site A, generally correlating with the qualitative detection of the *assA* gene (Table 2). One exception was for well C01-01, where the *assA* gene was not detected in the 2017 samples despite the detection of alkylsuccinates. In all sampled wells, alkylsuccinates were detected when the C_6_-C_10_ alkane concentrations were 0.1 ppm or greater. While neither the *assA* gene nor alkylsuccinates were detected in the majority of the non-contaminated wells (wherein alkanes were below the detection limit of <0.1 ppm), two anomalies were observed (Table 2). Two alkylsuccinates were detected in non-contaminated well C03-11 in both 2016 and 2017, although no *assA* genes could be qualitatively detected; for this well, it is possible that groundwater flow transported alkylsuccinates produced elsewhere in aquifer into the vicinity of the groundwater well. The *assA* gene was detected in well C02-07 in the 2016 sampling event, although corresponding metabolites were not found.

At Site B, fumarate addition metabolites derived from alkanes were detected in 10 of the 12 wells sampled (Table 2). The alkylsuccinates identified were primarily derived from C_5_ to C_7_ linear or cyclic alkanes. As with the results from Site A, a co-occurrence of alkylsuccinates and the *assA* gene (as determined qualitatively) was observed for the majority of the sample wells at Site B, with a few exceptions. Some groundwater samples revealed the presence of the *assA* gene, but not the corresponding alkylsuccinates (ISO-49 in 2016, REC 12 in 2017, REC 26 in 2016 and 2017). Alkylsuccinates nor the *assA* gene could be qualitatively detected at the lone non-contaminated well sampled at Site B, well S14-49B (Table 2). Despite the lack of universal co-occurrence of alkylsuccinates and the *assA* gene, the results on balance offer strong evidence that the microbial communities indigenous to the groundwater at both Sites A and B are capable of in situ anaerobic alkane biodegradation.

**Figure 5:**
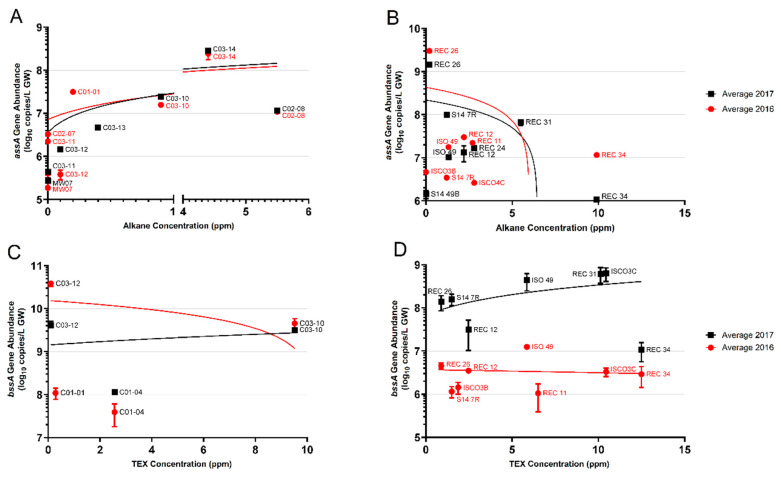
Quantifications of the *assA* and *bssA* genes in Sites A and B across 2 years (2016, red circles; 2017, black squares), plotted as a function of alkane or TEX (toluene, ethylbenzene, and xylene) concentrations, respectively. (**A**) *assA* gene abundances in Site A wells; (**B**) *assA* gene abundances in Site B wells; (**C**) *bssA* gene abundances in Site A wells; (**D**) *bssA* gene abundances in Site B wells. Linear regression trend lines are matched in colour. Only those samples whose amplicons matched the length of standard amplicons are plotted. Error bars represent the standard deviation of technical replicates (*n* = 3).

In addition to the presence/absence analysis of the *assA* gene based on PCR, we also quantified *assA* gene abundances using a mixture of newly designed qPCR primers (Figure 5, Table 2). Using these qPCR primers, the *assA* gene (verified by Sanger sequencing) was detected in 9 of the 12 wells at Site A (Figure 5A). At Site A, qPCR analyses showed that *assA* gene abundances increased from log 5–6 copies/L in non-contaminated groundwater wells to log 7–8 copies/L in wells with measurable alkane concentrations. Quantification of the *assA* gene showed that the highest alkane-contaminated wells, C02-08 (5.5 ppm total alkanes) and C03-14 (4.4 ppm total alkanes) had *assA* abundances of 10^7^copies/L and 10^8^ copies/L, respectively. The qPCR primer mixture revealed between 10^5^ and 10^6^ copies/L in three of the five wells with non-detectable alkane concentrations (wells C02-07, C03-11, and MW-07). In general, increased abundances of the *assA* gene positively correlated with increased alkane concentrations at Site A (Figure 5A).

Quantification of the *assA* gene in Site B samples showed a range of almost 4 orders of magnitude difference across the site (Figure 5B). Well S14-49B, the sole uncontaminated well, harboured 10^6^ copies/L of the *assA* gene, the lowest abundance measured at Site B. Of the 16 wells (both years) from which *assA* could be quantified, nine harboured 10^7^ copies/L, spanning alkane concentrations from approximately 2 ppm (REC 12) to 10 ppm (REC 34, 2016 sample only) (Figure 5B). However, the highest *assA* gene abundance of 10^9^ copies/L was found in well REC 26, where only 0.2 ppm alkanes were measured. In contrast, REC 34, the groundwater well having the highest measured alkane concentration of all the sampled wells (~10 ppm), had 10^6^ and 10^7^ *assA* gene copies/L, differing in an order of magnitude between sampling years (Figure 5B). The majority of the wells sampled at Site B harboured between 5 × 10^6^ and 10^8^ *assA* gene copies/L, with only a single well harbouring 10^9^ copies/L. At Site B, the *assA* gene abundance appears to increase with increasing alkane concentrations up to a limit of approximately 5 ppm alkanes; after this concentration, this trend is no longer seen. Additional samples with >5 ppm alkanes would be needed to verify this observation (Figure 5B).

#### 3.2.2. Benzylsuccinates and *bssA* Genes

Table 3 summarizes the fumarate addition metabolites and *bssA* genes detected from Sites A and B. The frequency of benzylsuccinate (or an alkylated analog) detection in all sampled wells was comparatively lower than for alkylsuccinates for both sites (Table 2). For site A, methylbenzylsuccinates were detected only in the two most highly contaminated wells (C02-08 and C03-14). For well C02-08, the metabolites and *bssA* gene (qualitative detection) co-occurred, while this was only the case in 2017 from well C03-14 (Table 2). Fumarate addition metabolites derived from alkylbenzenes were not detected in any other samples from this site, although *bssA* was present almost universally, including in the samples wherein alkylbenzenes were below detection.

A similar lack of alkyl aromatic metabolite detection was seen for Site B groundwater samples (Table 3). While a co-occurrence of benzylsuccinates and the *bssA* gene was evident in three samples (REC 34, ISO-49 in 2016, S14-7R in both years), this was not the case for the majority of the samples. Furthermore, for two contaminated groundwater samples at Site B (REC 31 and ISCO-04-C), neither a fumarate addition metabolite nor the *bssA* gene were detected using a qualitative approach (Table 3).

Figure 5C shows the quantification of the *bssA* gene plotted against TEX concentrations in Site A. False positives (those with non-specific amplicons) and zero value quantifications were not plotted. Only 4 wells had quantifiable *bssA* with the qPCR primers used. Wells C03-12 and C03-10 revealed the most abundant *bssA* per litre groundwater (C03-12 2016 = 10^10^ copies/L, 2017 = 10^9^ copies/L; C03-10 2016 and 2017 = 10^9^ copies/L). With this limited data, there was no correlation between TEX concentration and *bssA* gene copies, as wells C03-12 and C03-10 harboured a similar bssA gene abundance, despite a 10-fold difference in TEX concentrations (Figure 5C, Table 3).

Quantification of the *bssA* gene in Site B samples plotted against TEX concentration shows that the minimum abundance of *bssA*, regardless of TEX concentration, was approximately 1 × 10^6^ copies/L (Figure 5D). Only one well, S14-7R, had tested positive in both 2016 and 2017, which saw a 138-fold increase in *bssA* copies/L from 2016 to 2017. As with Site A, there did not appear to be any correlation between TEX concentrations and quantities of the *bssA* gene; wells with similar TEX concentrations exhibited at least 2 orders of magnitude difference in *bssA* abundance. Overall, the quantification of the *bssA* gene in the two field sites suggests that *bssA* gene abundances are independent of aromatic hydrocarbon concentrations using the designed qPCR primers.

### 3.3. *assA* and *bssA* Amplicon Sequencing Results

**Figure 6:**
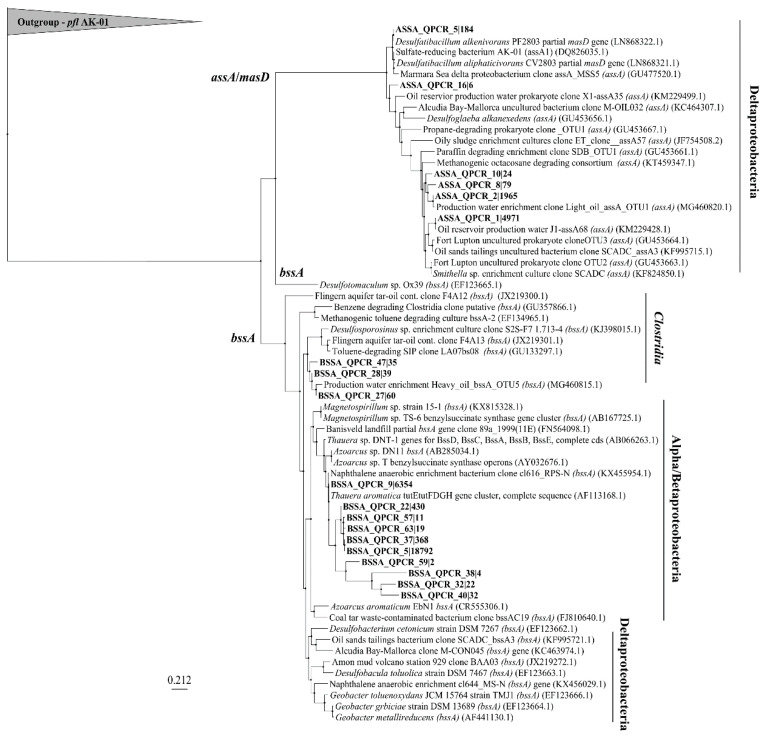
Maximum likelihood phylogram of sequenced *assA* and *bssA* amplicons. Nucleic acid sequences were aligned with ClustalX (30 iterations). Tree-building via PhyML 3.0 Maximum Likelihood using TN93 substitution model, fast likelihood-based method aLRT (approximate likelihood ratio test) for branch supporting, and SPR (subtree pruning and re-grafting) tree improvement model. The outgroup is pyruvate formate lyase (pfl) from sulfate-reducing strain *D. alkenivorans* strain AK-01.

Figure 6 shows the relationship of sequenced *assA* and *bssA* amplicons present in the site groundwaters following amplification of these genes with the newly designed qPCR primers. After read preparation and quality control of amplicons, a BLASTn search of 86 sequences returned 6 that matched NCBI entries annotated as *assA* with >70% identity and coverage. Clustering of the various sequences shows that 2 main clusters of *assA* sequences were present in these samples, all within the deltaproteobacteria; those that affiliated with sulfate-reducers (such as *Desulfatibacillum alkenivorans*, and *Desulfatibacillum aliphaticivorans*) and those affiliating with methanogenic/syntrophic cultures (with *Smithella* sp.) or oilfield-derived clones [19,22,46,47]. Some BLASTn results affiliated with the *assA* genes of the organisms from which the primers were designed. For example, primer ‘assOri’ was designed from *D. alkenivorans,* and primer ‘assSml’ was designed to capture *Smithella* SCADC sequences; sequences closely related to the *assA* genes from these taxa were found (Figure 6). The other primers in the primer mix, ‘assMsd’ and ‘assEx’ were designed from an annotated *masD* sequence and *Desulfoglaeba alkenexedens*, respectively. An *assA* sequence similar to that of *D. alkenexedens* was detected in these samples. It is likely that the directed design of the primers in the primer mix influenced the types of *assA* sequences that resulted from the analysis. Many of the BLASTn matches were not annotated as *assA* yet had coverage and identity above 70%. These were instead annotated to be involved in protein catabolism, ribosomes, or other metabolic functions (Table S4). Many of the organisms harbouring the sequence mismatches are known as aerobic/microaerophilic and are present in hydrocarbon-contaminated sites such as *Variovorax* (aerobic, aromatics), *Polaromonas* (aerobic, aromatics), *Azoarcus* (facultative, aromatic and alkane), *Massilia* (aerobic, PAH), and others [48,49,50,51]. This suggests that either the *assA* gene is present in these genomes but is currently uncharacterized, or non-specific binding is amplifying non-*assA* genes from these organisms, or many sequences annotated in silico may not be annotated as FAE genes. Notably, this non-specific binding explanation has an additional consequence of inflating gene abundances in quantification assays, which we cannot rule out here. Previous groups have not used next-generation sequencing platforms for their *assA* or *bssA* identifications, and thus such non-specific binding has not been reported [19,20,22,23,40]. Future work that comprehensively examines the possible explanations for sequence mismatches (that includes additional sequences retrieved from other sites) is warranted.

For the *bssA* gene, a BLASTn search yielded 13 OTUs (operational taxonomic units) with matches to *bssA* sequences from 65 quality assessed sequences. A maximum likelihood analysis shows that 2 main clusters of *bssA* formed, one clustering within the Clostridia, and the other clustering within the *Alphaproteobacteria* and *Betaproteobacteria*, such as *Thauera* (*tut* operon) [52], *Azoarcus*, and *Magnetospirillum*. No *bssA* sequences were retrieved from *Deltaproteobacteria*. The *tut* operon found in *Thauera aromatica* was also a dominant match in many of the sequences. Such clustering across betaproteobacterial species like *Azoarcus* and *Thauera*, suggests that these sequences are very similar, and are also similar to *Magnetospirillum* spp. *bssA* sequences. Compared to non-specific matches using the *assA* primer mix, few *bssA* non-matches were seen. These included matches to an acyl-CoA dehydrogenase (which is involved in β-oxidation of fatty acids), a 30S rRNA gene (smaller subunit of prokaryotic ribosome), and surprisingly, an *assA* match.

## 4. Discussion

A multitude of approaches can be used to determine whether a hydrocarbon-contaminated groundwater site is undergoing bioremediation, each with advantages and limitations [25]. For example, measuring concentration differences of potential electron acceptors (such as O_2_, nitrate, sulfate) or respiration products (such as CO_2_, Fe(II), sulfide, CH_4_), or changes in microbial numbers and/or taxonomic compositions in contaminated areas versus uncontaminated areas can indicate microbial changes in response to hydrocarbon presence [25,26]. However, these measurements do not directly indicate that hydrocarbons are being biodegraded in situ. Compound-specific isotope analysis (CSIA) which measures isotopic changes in hydrocarbons themselves as they are being biotransformed, is a powerful approach for proving biodegradation, but isotopic fractionation can vary in heterogeneous sites and large isotopic changes are needed [25]. Similarly, identifying known hydrocarbon metabolites in hydrocarbon-contaminated groundwater provides direct evidence that the extant microorganisms are indeed biotransforming hydrocarbons in situ [5,27], but requires prior knowledge of such degradation pathways. Thus, coupling multiple approaches to assess sites for their bioremediation potential can help overcome methodological limitations. In this study, two hydrocarbon-contaminated groundwater sites, candidates for management by MNA, were interrogated using measurements of biogeochemical indicators, microbial community profiling, and diagnostic hydrocarbon metabolites and genes in order to garner evidence in support of in situ hydrocarbon biodegradation.

While biogeochemical measurements indicated that nitrate, sulfate, and/or Fe(III) reduction may be operating at both sites, no clear trends were seen that suggested any dominant electron accepting process occurring at either site. Similarly, microbial community profiling based on the 16S rRNA gene indicated the presence of several anaerobic taxa and/or putative hydrocarbon-degraders, but taxonomic variation between all sampled groundwater wells precluded any clear conclusions that hydrocarbon-biodegrading taxa may have been stimulated in one site location versus another. Of note, both H_2_- and acetate-using methanogens were also identified via 16S rRNA gene sequencing, with the highest relative sequence abundance found in the uncontaminated well sampled at Site B. Although H_2_ and acetate were not measured in this study, we can speculate that they were formed in uncontaminated groundwater during the decomposition of dissolved organic matter by syntrophic bacteria acting in concert with methanogens. Previous reports describing microbial communities and biogeochemical transformations in pristine groundwater have suggested such interactions stimulated by dissolved organic matter decomposition [53,54].

Fumarate addition metabolites known to originate from the anaerobic biodegradation of both alkanes and alkyl monoaromatics were detected at several locations at both sites. The corresponding diagnostic biodegradation genes, *assA* and *bssA*, were also widely detected at both sites. Collectively, these results clearly demonstrated that both sites harbour anaerobic hydrocarbon-degrading microbial populations; as such, MNA could be a remedial option at these sites. Fumarate addition metabolites have been detected in many hydrocarbon-contaminated groundwater aquifers [5]. Most previous studies have focused on the identification of (methyl)benzylsuccinates originating from alkyl-substituted monoaromatic hydrocarbons, with fewer reports describing the detection of alkylsuccinates derived from alkanes in contaminated groundwater environments [36,55,56]. Refined fuel mixtures, such as the gasoline and diesel contaminants relevant to this study, are composed of low molecular weight alkanes and cyclic alkanes in addition to monoaromatic hydrocarbons. Thus, detecting fumarate addition metabolites from both classes of hydrocarbons is not unexpected in groundwater systems contaminated with such fuels [36,55].

The *bssA* gene, encoding the FAE benzylsuccinate synthase, has also been sought and detected in numerous groundwater sites as an indicator of hydrocarbon biodegradation and bioremediation potential [19,21,22,29,30,31,32,57,58]. In contrast, the *assA* gene (encoding alkylsuccinate synthase) has rarely been used to interrogate hydrocarbon-contaminated groundwater environments [20], although it is being increasingly sought and detected in marine systems such as hydrocarbon seeps and sediments [22,24,31,59]. We also detected both of these genes in many of the groundwater sampling locations at both sites under study, augmenting the metabolite findings (Table 2 and Table 3). With two exceptions (Site A, well C02-07 and Site B, well ISCO-03-B), the *assA* gene was detected only in wells also having detectable alkane concentrations (Table 2). In contrast, the *bssA* gene was detected in several wells having no detectable TEX concentrations (Table 3). Gittel et al. [23] detected *assA* genes in both pristine and alkane-containing marine seep samples, suggesting the ubiquity of such genes and supporting the notion that pristine environments naturally harbor microorganisms capable of hydrocarbon metabolism when exposed to hydrocarbons. Such a hypothesis may pertain to terrestrial groundwater environments as well, in light of the findings in this present study and that by Brow et al. [60]. These latter authors reported *bssA* transcripts on the order of log 4–5 /mL in groundwater samples showing no detectable toluene, suggesting a basal/constitutive level of *bssA* expression in groundwater-associated microbial communities.

Although we observed a co-occurrence of both fumarate addition metabolites and relevant genes in many of the groundwater wells sampled for this study, this was not always the case. Alkylsuccinates were detected in parallel with qualitative *assA* gene detection in most wells (Table 2), although only one of these diagnostic indicators was detected in a small number of samples. The simultaneous detection of both (methyl)benzylsuccinate and the *bssA* gene was much less pronounced than for the alkane degradation indicators. At Site A, only 2 of the 12 sampled wells showed the detection of both relevant metabolite and gene, only 3 of the 12 wells at Site B showed a co-occurrence of a (methyl)benzylsuccinate and the *bssA* gene. Such discrepancies were also noted in other studies that analyzed for both the *bssA* gene and related metabolites [30,32], where only one or the other diagnostic indicator was detected in many sampled wells. While the reasons for these disparities are not completely clear, some explanations can be considered. For example, the lack of detection of a given diagnostic metabolite in a contaminated groundwater system does not necessarily mean that the microbial community does not have the ability to biodegrade the parent hydrocarbon. As fumarate addition metabolites are metabolic intermediates, they may not accumulate to levels above that of the analytical detection limits, thus preventing their identification. Furthermore, as these metabolites have limited solubility at the low concentrations in which they may be detected (nM levels), it is also possible that they were transported in groundwater away from the location where they were initially produced and thus were not detected in the sampled locations. This may be especially true for benzylsuccinates which were not detected in the majority of the wells. However, because the relative aqueous solubilities of benzylsuccinates versus alkylsuccinates are not known, this explanation remains speculative. Similarly, metabolite assays as conducted here represent a ‘snapshot’ of the groundwater chemistry at the time of sampling [36,55], and thus the activity of the microbial community, along with dilution effects due to groundwater flow, can impact the positive detection of metabolites such as benzylsuccinates.

Similarly, the lack of detection of *bssA* or *assA*, despite metabolite detection, may be due to limitations with the primer sets used for their amplification and detection. Both of these genes are known to be highly diverse, spanning multiple taxa and environments [22,23,24]. As such, no single primer pair designed to date has captured *assA* or *bssA* diversity comprehensively [19,20,29,30]. Such may be the case in the present study, where a single primer set designed for each gene was used for its qualitative detection.

Owing to the limitation in diversity capture, few primer sets have been designed for the quantification of *assA* or *bssA* genes. Those that have been designed have a limited detection scope, either specific for sulfate-reducing or nitrate-reducing strains or for taxa in specific enrichments [22,23,40,60,61]. For example, widely used qPCR *bssA* primers were designed exclusively from betaproteobacterial *bssA* sequences, namely from *Thauera aromatica* strains K172 and T1, and *Azoarcus* sp. strain T [61]. As such, the sequences from other phyla may not be detected, an acknowledged limitation [61]. Other primer sets were subsequently designed to target additional taxa such as sulfate-reducing members of the *Deltaproteobacteria* and *Clostridia* [19,22]. Stagars et al. [24] designed 4 fluorescence FISH (fluorescence in-situ hybridization) probes which would allow for quantification of different clusters of *assA* sequences identified in their study examining this gene in marine environments, however the prevalence of non-specific probe annealing reinforced the difficulty in accurately detecting FAE genes. Using the qPCR primer set designed here and sequencing the resulting amplicons, we positively identified *bssA* genes affiliating with the *Clostridia, Alphaproteobacteria,* and *Betaproteobacteria* and *assA* genes associated with organisms in the *Deltaproteobacteria*.

The qPCR primers newly designed for this study used a mixture of 4 forward primers and 1 reverse primer for each of the *assA* and *bssA* genes (Table 1). To our knowledge, this primer mixture approach, designed to capture broad diversity (see Supplementary Materials), has not been previously attempted for *assA* nor *bssA* gene quantification. Using the qPCR primer mixture, we obtained gene abundances approximately 2 orders of magnitude greater than any other gene abundance value reported by other groups (Table 4). Generally, in contaminated aquifers, the gene abundance of *bssA* genes has been found to be between 10^3^ to 10^8^ copies/g sediment or copies/L water (Table 4). The large difference in the abundances of the *bssA* gene, ranging across 5 orders of magnitude, may also be dependent upon experimental differences, such as the primers used, the type of sample, toluene degradation activity, and the extent to which BSS-harbouring microorganisms were enriched in laboratory cultures or groundwater environments. Studies relating the abundance of *bssA* to 16S rRNA gene abundances found that while the 16S rRNA gene abundances were relatively consistent, the abundances of the *bssA* genes varied across orders of magnitude [30]. Other groups have detected background abundances (e.g., in uncontaminated samples) of *bssA* to be approximately 10^4^ copies/L, while the lowest abundance captured in this study was 10^5^ copies/L (Table 4). With this higher detection limit, we may not have adequately quantified the *bssA* gene, especially in the Site A samples where we were not able to quantify this gene in many samples even though it was detected using the qualitative PCR assay (Table 3). Based on the limited number of wells wherein the *bssA* gene was quantified, no relationships between the *bssA* gene abundances and TEX concentrations were discernible.

In comparison with the few reported *assA* quantification studies, the overall abundances are similar, up to ~8 log per unit sample in higher contaminated samples (Table 4). To our knowledge, very few studies have quantified *assA* gene abundances in environmental samples, much less than for *bssA* (Table 4), thus it is not known how our results compare to other field sites. Further studies of *assA* gene abundances in contaminated groundwater environments are needed. In contrast to *bssA* and TEX concentrations, there did appear to be a relationship between *assA* gene abundances and alkane concentrations wherein *assA* levels increased as alkane concentrations increased, at least up to 5 ppm alkanes. However, many more samples would need to be analyzed across many different sites to verify this trend.

The overall results using the newly designed qPCR primers for *assA* and *bssA* showed that they did capture a diversity of taxa within the *Alphaproteobacteria*, *Betaproteobacteria*, *Deltaproteobacteria*, and *Clostridia*, offering a proof-of-concept demonstration of a customized primer mix for quantifying FAE genes in a groundwater environment. These data suggest that the use of a primer mix to target specific diversity can work, if prior knowledge allows a directed and purposeful design. As additional *assA* and *bssA* gene sequences become available in databases, adding these to and/or further optimizing the primer mix should aid in improved amplification and quantification of these indicative biodegradation genes in hydrocarbon-associated environments.

## 5. Conclusions

Analyses of two hydrocarbon-contaminated groundwater systems, focused on combining assays for signature metabolite detection and FAE gene detection and quantification, revealed that both sites investigated showed strong potential for management by MNA. Newly designed qPCR primer mixtures revealed up to 10^9^ *assA* gene copies/L and up to 10^10^ *bssA* gene copies/L at the study sites. While previous studies have reported the quantification of the *bssA* gene in hydrocarbon-contaminated groundwater, this present study has now also demonstrated the detection and quantification of *assA* genes in groundwater systems. While both metabolites and genes indicative of in situ hydrocarbon biodegradation were detected in some of the wells investigated, this was not always the case, similar to previous reports [30,32]. Thus, assessing field sites for evidence of in situ anaerobic hydrocarbon biodegradation should minimally use both approaches (in addition to other site assessments) to overcome any limitations each method may have in order to demonstrate the potential for hydrocarbon-contaminated groundwater to be naturally remediated by microorganisms.

## Figures and Tables

**Table 1:** Primers designed and used in this study for quantitative detection of the *bssA* and *assA* genes in Site A and B groundwater samples. Melting temperature T_m_ was calculated via the IDT OligoAnalyzer 3.1 online tool using default quantitative polymerase chain reaction (qPCR) parameters.

Name	Sequence (5’-3’)	Tm (°C)	Reference
***bssA forward***			
bssOil	GAA TCC CTG GTT ACA GGT CCA C	64.1	This study
bssMys	CAA TCC GTG GCA CAA CTG CAT G	66.3	This study
bssSuf	GAA TAC GTG GAG CGA CCC GCT C	68.1	This study
bssWin	CAA TCC GTG GCT TCA GGT TCA T	65	This study
***bssA reverse***			
bssHitr	TCC TCG TAG CCT TCC CAG TT	64.6	This study
***assA forward***			
assOri	CTC CGC CAC GGC CAA CTG	67.4	This study
assMsd	CTC AGC CAC CGC CAA CTG	65	This study
assEx	CTC TGC GAC CGC GAA TTG	63	This study
assSml	TAG CGC CAC GGC CAA CTG	67	This study
***assA reverse***			
8543r	TCG TCR TTG CCC CAY TTN GG	65.7	[22]

**Table 2:** Detection of alkane/cyclic alkane-derived fumarate addition metabolites and *assA* genes (qualitatively and quantitatively determined) in Site A and B groundwater wells sampled in 2016 and 2017. “2016” or “2017” indicates that the metabolite was detected in that year, while “*assA*” denotes gene detection. A blank space denotes absence of metabolite or gene.

		Alkanes	Detected Alkylsuccinates										*assA* Gene Presence		*assA* Gene Abundance (copies/L GW) ^5^	
	Well	C6-C10 Alkanes (ppm)	C5	C5 unsat ^1^	C6	C6 unsat	C7	C7 unsat	C8	C8 unsat	C9	C9 unsat	2016	2017	2016	2017
**Site A**	C02-08	5.5		2016		2016	2017		2016/2017	2016	2016		*assA*	*assA*	1.09 × 10^7^	1.17 × 10^7^
	C03-14	4.4				2016		2016/2017		2016		2016	*assA*	*assA*	2.43 × 10^8^	2.86 × 10^8^
	C03-10	0.9	2016/2017	2016		2016/2017		2016/2017		2016/2017		2017	*assA*	*assA*	1.57 × 10^7^	2.45 × 10^7^
	C01-04	0.5	2016/2017			2016/2017		2016/2017		2016			*assA*	*assA*		
	C03-13 ^3^	0.4					2017		2017			2017		*assA*	-	4.64 × 10^6^
	C01-01	0.2				2016	2016/2017		2016/2017	2016/2017	2016/2017	2016/2017	*assA*		3.15 × 10^7^	
	C03-12	0.1						2016		2016		2017	*assA*	*assA*	3.81 × 10^5^	1.45 × 10^6^
	C02-06	<0.1 ^2^														
	C02-07	<0.1											*assA*		3.27 × 10^6^	
	C03-11	<0.1				2016/2017		2016/2017							2.25 × 10^6^	4.34 × 10^5^
	MW 07	<0.1													1.86 × 10^5^	2.72 × 10^5^
	MW23	<0.1														
	TRIP BLANK	<0.1														
**Site B**	REC-34	9.9	2016/2017	2016/2017		2016/2017		2016					*assA*	*assA*	1.15 × 10^7^	1.08 × 10^6^
	REC-31	5.5	2017	2016/2017		2016/2017		2016/2017						*assA*		6.61 × 10^7^
	ISCO-03-C	3.1	2016	2016		2016						2016	*assA*	*assA*		
	ISCO-04-C ^4^	2.8	2016	2016		2016							*assA*		2.61 × 10^6^	-
	REC-24	2.8	2016/2017	2016/2017		2016/2017							*assA*	*assA*		1.69 × 10^7^
	REC-11 ^4^	2.7	2016	2016									*assA*		2.21 × 10^7^	-
	REC-12	2.2	2016				2016						*assA*	*assA*	3.00 × 10^7^	1.34 × 10^7^
	ISO-49	1.3	2017		2017	2017		2017					*assA*	*assA*	1.76 × 10^7^	1.03 × 10^7^
	S14-7R	1.2	2016/2017	2016/2017		2016/2017		2017					*assA*	*assA*	3.44 × 10^6^	9.80 × 10^7^
	REC-26	0.2											*assA*	*assA*	3.02 × 10^9^	1.45 × 10^9^
	ISCO-03-B ^4^	<0.1				2016							*assA*		4.64 × 10^6^	-
	S14-49B	<0.1													1.36 × 10^6^	1.42 × 10^6^
	Trip Blank	<0.1														

^1^ unsat indicates a fumarate addition product with 2 mass units less that an *n*-alkane, presumably derived from the metabolism of a cyclic alkane; ^2^ below the analytical detection limit of 0.1 ppm; ^3^ well not sampled in 2016; ^4^ well not sampled in 2017; ^5^ A dash indicates that no quantification was performed as no sample was received that year. A blank space indicates that the gene could not be detected or quantified using the quantitative polymerase chain reaction (qPCR) primer set.

**Table 3:** Detection of TEX-derived fumarate addition metabolites and *bssA* genes (qualitatively and quantitatively determined) in Site A and B groundwater wells sampled in 2016 and 2017. “2016” or “2017” indicates that the metabolite was detected in that year, while “*bssA*” denotes gene detection. A blank space denotes absence of metabolite or gene.

		Monoaromatic Hydrocarbon			Benzylsuccinates			*bssA* Gene Presence		*bssA* Gene Abundance (copies/L GW) ^4^	
	Well	Toluene (ppm)	Ethylbenzene (ppm)	Xylenes (ppm)	BzSucc	EBSucc	MeBzSucc	2016	2017	2016	2017
**Site A**	C02-08	0.072	0.861	1.71			2016/2017	*bssA*	*bssA*		
	C03-14	0.0976	1.8	0.0102			2016/2017		*bssA*		
	C03-10	0.128	1.26	0.178				*bssA*	*bssA*	4.48 × 10^9^	3.18 × 10^9^
	C01-04	0.0153	0.0912	0.0081				*bssA*	*bssA*	3.90 × 10^7^	1.13 × 10^8^
	C03-13 ^2^	<0.0003	0.013	0.0102							
	C01-01	0.0014	<0.0005	0.001				*bssA*		1.09 × 10^8^	
	C03-12	<0.0003	0.0018	0.0015				*bssA*	*bssA*	3.84 × 10^10^	4.08 × 10^9^
	C02-06	<0.0003	<0.0005	<0.0005							
	C02-07	<0.0003	<0.0005	<0.0005				*bssA*			
	C03-11	<0.0003	<0.0005	<0.0005				*bssA*	*bssA*		
	MW07	<0.0003	<0.0005	0.0102				*bssA*			
	MW23	<0.0003	<0.0005	0.0102				*bssA*	*bssA*		
	TRIP BLANK	<0.0003 ^1^	<0.0005 ^1^	<0.0005							
**Site B**	REC-34	3.83	0.325	6.11	2016/2017	2016	2016/2017	*bssA*	*bssA*	2.89 × 10^6^	1.06 × 10^7^
	REC-31	0.867	0.394	0.979							6.17 × 10^8^
	ISCO-03-C	0.557	0.0051	0.0051				*bssA*	*bssA*	3.30 × 10^6^	6.29 × 10^8^
	ISCO-04-C ^3^	0.92	0.0323	0.0323							
	REC-24	0.377	0.297	0.675				*bssA*	*bssA*		
	REC-11^3^	0.732	0.0408	4.96				*bssA*		1.05 × 10^6^	-
	ISO-49	0.87	0.223	4.46	2016		2016	*bssA*	*bssA*	1.24 × 10^7^	4.37 × 10^8^
	REC-12	0.0299	0.0489	0.0432					*bssA*	3.51 × 10^6^	3.11 × 10^7^
	S14-7R	0.125	0.0365	0.0754	2016/2017			*bssA*	*bssA*	1.15 × 10^6^	1.59 × 10^8^
	ISCO-03-B ^3^	0.0713	0.108	0.0454				*bssA*		1.44 × 10^6^	-
	REC-26	0.0523	0.0421	0.403					*bssA*	4.53 × 10^6^	1.38 × 10^8^
	S14-49B	<0.0003	<0.0005	<0.0005					*bssA*		
	Trip Blank	<0.0003	<0.0005	<0.0005							

^1^ below the analytical detection limit of 0.0003 ppm for toluene and 0.0005 ppm for ethylbenzene or xylenes; ^2^ well not sampled in 2016; ^3^ well not sampled in 2017; ^4^ A dash indicates that no quantification was performed as no sample was received that year. A blank space indicates that the gene could not be detected or quantified using the qPCR primer set.

**Table 4:** Comparisons of approximate reported *assA* and *bssA* gene abundances reported in literature and in the present study. Note that abundances reported in this study are up to 2 log greater than other studies, in both detection limit and in highest reported abundances.

Targeted FAE Gene	Reported Detection Limit (copies/L or g)	Highest Reported Sample Abundance (copies/L or g)	Targeted Electron-Accepting Conditions	Cited Primer Reference	Reference
*assA*	~log 5	~log 9	Sulfate-reducers and methanogenic consortia		This Study
	not reported	~log 6	Methanogenic paraffin degrading consortium		Oberding and Gieg [62]
	~log 3	~log 8	Sulfate-reducers and methanogenic consortia		Aitken et al. [40]
					
*bssA*	~log 5	~log 10	Nitrate- and sulfate-reducers		This Study
	~log 2	~log 8	Sulfate-reducers	Winderl et al. [29]	Pilloni et al. [63]
	~log 4	~log 8	Nitrate- and sulfate-reducers	Winderl et al. [19] non-qPCR primers 7772f/8546r	Müller et al. [64]
	~log 4	~log 4	Sulfate-reducers and methanogenic consortia	Beller et al. [30]	Oka et al. [32]
	~log 2	~log 3	Nitrate-reducers	Beller et al. [61]	Oka et al. [32]
	~log 1	~log 8	Nitrate-reducers	Beller et al. [61]	Kazy et al. [57]
	~log 3	~log 8	Sulfate-reducers		Beller et al. [30]
	~log 3	~log 7	Deltaproteobacterial “F1”		Winderl et al. [29]
	~log 2	~log 8	Nitrate-reducers	Beller et al. [61]	Da Silva and Alvarez [58]
	~log 3	~log 8	Nitrate-reducers		Beller et al. [61]

## Supplementary Materials

The following are available online at https://www.mdpi.com/2076-2607/8/10/1532/s1: Supplementary Methods: Design of *assA* and *bssA* primer mixture, Table S1: Total hydrocarbon concentrations in groundwater collected from various wells from Site A and Site B, Table S2: Sequences used in *assA* and *bssA* primer design with description and accession number from NCBI database, Table S3: Designed Illumina MiSeq adapter primers for *assA* and *bssA* qPCR primers, Table S4: Examples of sequenced *assA* assay products that passed quality control analysis but that were not annotated as alkylsuccinate synthase genes using a BLASTn analysis.
