# Supplementary material for: Combined Use of Diagnostic Fumarate Addition Metabolites and Genes Provides Evidence for Anaerobic Hydrocarbon Biodegradation in Contaminated Groundwater
Source: Microorganisms. 2020 Oct 6;8(10):1532. doi: 10.3390/microorganisms8101532 (PMC7599786; doi:10.3390/microorganisms8101532)
Supplement: Supplementary file 1 [file microorganisms-08-01532-s001.pdf]

Supplementary Materials

Combined use of diagnostic fumarate addition metabolites and genes provides evidence for anaerobic hydrocarbon biodegradation in contaminated groundwater

Gurpreet Kharey[1], Gabrielle Scheffer, and Lisa M. Gieg*

Department of Biological Sciences, University of Calgary, 2500 University Drive NW, Calgary, AB T2N 1N4, Canada; gurpreet.kharey@mail.mcgill.ca; gabrielle.scheffe1@ucalgary.ca

* Correspondence: lmgieg@ucalgary.ca; Tel.: 1-403-210-7207

[1]Present address: Department of Natural Resource Sciences, McGill University, 21111 Lakeshore Road, Sainte-Anne-de-Bellevue, QC H9X 3V9/National Research Council of Canada, 6100 Royalmount Ave, Montreal, QC H4P 2R2, Canada

Supplementary Methods
Supplementary Tables

Supplementary Methods

Design of *assA* and *bssA* primer mixture

Searching for '*assA*' and 'alkylsuccinate synthase' in the NCBI database resulted in ~1,700 entries, from which 20 were selected (7 from organismal entries, 9 from non-taxonomic clones). Similarly, a search for '*bssA*' and 'benzylsuccinate synthase' resulted in ~16,000 entries, from which 17 sequences were selected (9 from organismal entries, 8 from non-taxonomic clones) (Table S2). Initially a larger pool of sequences were selected from the NCBI database, in an attempt to encompass different sampling sites and research groups, but were removed due to repeated sequences.

Selected sequences were compiled into a Multiple Sequence Alignment (MSA) using ClustalOmega aligning algorithm via the Benchling online tool, replacing duplicated sequences [1, 2]. Regions of sequence homology were identified (i.e. regions that had fewest basepair mismatches), and forward primers were designed using the built-in Primer3 function. The primers generated by Primer3 did not align with the selected regions to target, thus they were adjusted slightly in length to bind to the intended region. This was done to ensure uniform produced amplicons, which would otherwise complicate qPCR analyses. Primers specific to the differing sequences in the MSA were made by modifying the new primer to be identical to sequences in the MSA that were not homologous with each other. The *assA* forward primer 'assOri' was designed from *D. alkenivorans* (Ori = Original; *D. alkenivorans* was set as the 'archetypal' organism)[3], and primer 'assSml' was designed to capture *Smithella* SCADC sequences (Sml = *Smithella*). The other primers in the primer mix, 'assMsd' and 'assEx' were designed from an annotated *masD* sequence and *Desulfoglaeba alkenexedens*, respectively (Table S2).

The *bssA* forward primers 'bssMys', 'bssWin', and 'bssOil' were designed from uncultured sequences (in NCBI: Mys = von Netzer submitted sequence, Win = Winderl submitted sequence, Oil = Uncultured sequence sample name with 'OIL003'). The lack of characterized *bssA* sequences aligning to specific genera hinders direct targeting of 'under-represented' *bssA* in mixed communities. Primer bssSuf was designed to target characterized taxa Suf = *Desulfotomaculum*) (Table S3).

The reverse primer used for *assA* was taken from literature, as sequence coverage in the MSA did not extend beyond the binding region of 8543r (the reverse primer used) [4]. The reverse primer for *bssA* was designed similarly to forward primers (as literature primers binding site was not reflected in the MSA across majority of sequences) and named bssHitr, which binds 48 bp upstream of 8546r (reverse primer[4]), and so named as it 'Hits' (aligns with) the upstream region. The amplicons derived from these primers were designed in such a way as to amplify a region *within* the amplicon from primers designed by Winderl et al. and von Netzer et al. as a method to ensure accurate amplification [4, 5].

Homo/hetero-dimerization among forward primers and the reverse primer was tested *in silico* using IDT OligoAnalyzer 3.1, discarding/redesigning any primer with $\Delta G < -7$ kJ. Forward primers were then combined into an equimolar (20 nM) mix (separate mixtures were prepared for *assA* and *bssA*). Primer mixtures were then tested via PCR no-template controls and positive controls using gDNA extracts from known *assA* and *bssA* harbouring isolates to optimize thermocycling conditions and reduce primer dimerization.

Supplementary Tables

Table S1. Total hydrocarbon concentrations in groundwater collected from various wells from Site A and Site B. Shading denotes concentrations above Alberta guidelines for allowable hydrocarbon concentrations in non-drinking groundwater [6].

	Well Name	Benzene (ppm)	Toluene (ppm)	Ethylbenzene (ppm)	Xylenes (ppm)	C_6 - C_{10} Alkanes (ppm)	Total Hydrocarbon (ppm)
	Guideline	0.005	0.002	0.002	0.02	2.2	
Site A	C01-01	0.3	0.0	<0.0005	0.0	0.2	0.5
	C01-04	2.5	0.0	0.1	0.0	0.5	3.1
	C02-06	<0.0005	<0.0003	<0.0005	<0.0005	<0.1	<0.1
	C02-07	<0.0005	<0.0003	<0.0005	<0.0005	<0.1	<0.1
	C02-08	2.2	0.1	0.9	1.7	5.5	10.3
	C03-10	8.0	0.1	1.3	0.2	0.9	10.4
	C03-11	0.1	<0.0003	<0.0005	<0.0005	<0.1	0.1
	C03-12	0.1	<0.0003	0.0	0.0	0.1	0.2
	C03-13	0.0	<0.0003	0.0	0.0	0.4	0.4
	C03-14	0.2	0.1	1.8	0.0	4.4	6.5
	MW07	<0.0005	<0.0003	<0.0005	0.0	<0.1	<0.1
	MW23	<0.0005	<0.0003	<0.0005	0.0	<0.1	<0.1
	Trip Blank	<0.0005	<0.0005	<0.0005	<0.0005	<0.1	<0.1
Site B	ISCO-3-B	1.7	0.1	0.1	0.0	< 0.1	3.8
	ISCO-3-C	9.9	0.6	0.0	0.0	3.1	23.9
	ISCO-4-C	9.3	0.9	0.0	0.0	2.8	23.2
	ISO 49	0.3	0.9	0.2	4.5	1.3	13.2
	REC 11	0.8	0.7	0.0	5.0	2.7	15.7
	REC 12	2.4	0.0	0.0	0.0	2.2	7.2
	REC 24	8.4	0.4	0.3	0.7	2.8	22.2
	REC 26	0.4	0.1	0.0	0.4	0.2	2.0
	REC 31	7.9	0.9	0.4	1.0	5.5	25.7
	REC 34	2.2	3.8	0.3	6.1	9.9	34.9
	S14-49B	<0.0005	<0.0003	<0.0005	<0.0005	< 0.1	<0.1
	S14-7R	1.3	0.1	0.0	0.1	1.2	4.2
	Trip Blank	<0.0005	<0.0003	<0.0005	<0.0005	< 0.1	<0.1

Table S2. Sequences used in *assA* and *bssA* primer design with description and accession number from NCBI database.

assA sequences (as per NCBI)	Accession Number	*bssA* sequences (as per NCBI)	Accession Number
Desulfatibacillum alkenivorans AK-01, complete genome	CP001322.1	*Desulfobacula toluolica* Tol2 complete genome	FO203503.1
Sulfate-reducing bacterium AK-01 alkylsuccinate synthase (assA1) gene, complete cds	DQ826035.1	*Geobacter metallireducens* benzylsuccinate synthase alpha subunit (bssA) gene, partial cds; and benzylsuccinate synthase beta subunit (bssB) gene, complete cds	AF441130
Sulfate-reducing bacterium AK-01 alkylsuccinate synthase (assA2) gene, complete cds	DQ826036	*Thauera aromatica* tdiSR and bssDCAB operons for benzylsuccinate synthase and a two-component regulatory system	AJ001848.3
Desulfoglaeba alkanexedens alkylsuccinate synthase (*assA*) gene, complete cds	GU453656.1	*Geobacter metallireducens* GS-15, complete genome	CP000148.1
Uncultured prokaryote clone Passaic_River_OTU2 alkylsuccinate synthase (assA) gene, partial cds	GU453657	*Thauera* sp. MZ1T, complete genome	CP001281.2
Uncultured prokaryote clone Gowanus_Canal_OTU1 alkylsuccinate synthase (assA) gene, partial cds	GU453659	*Desulfobacula toluolica* strain DSM 7467 benzylsuccinate synthase alpha subunit (bssA) gene, partial cds	EF123663
Uncultured prokaryote clone Fort_Lupton_OTU3 alkylsuccinate synthase (assA) gene, partial cds	GU453664	*Desulfotomaculum* sp. Ox39 benzylsuccinate synthase alpha subunit (bssA) gene, partial cds	EF123665.1
Uncultured prokaryote clone Arthur_Kill_OTU1 alkylsuccinate synthase (assA) gene, partial cds	GU453666	Sulfate-reducing bacterium TRM1 benzylsuccinate synthase alpha subunit (bssA) gene, partial cds	EF123667
Uncultured bacterium clone BGM02 alkylsuccinate synthase alpha subunit (assA) gene, partial cds	JX219367	Uncultured bacterium clone B49bss_021 benzylsuccinate synthase alpha subunit (bssA) gene, partial cds	EF123670
Uncultured bacterium clone BGM24 alkylsuccinate synthase alpha subunit (assA) gene, partial cds	JX219368	Uncultured bacterium clone Pb312_80 benzylsuccinate synthase alpha subunit (bssA) gene, partial cds	EF123703
Uncultured bacterium clone M-NAPH012 alkylsuccinate synthase alpha subunit (assA) gene, partial cds	KC464263.1	Uncultured prokaryote clone Fort_Lupton_OTU1 benzylsuccinate synthase (bssA) gene, partial cds	GU453672
Uncultured bacterium clone M-OIL045 alkylsuccinate synthase alpha subunit (assA) gene, partial cds	KC464317	Uncultured bacterium clone F3A10 benzylsuccinate synthase alpha subunit (bssA) gene, partial cds	JX219282.1
Smithella sp. enrichment culture clone SCADC alkylsuccinate synthase alpha subunit (assA) gene, complete cds	KF824850	Uncultured bacterium clone F5A30 benzylsuccinate synthase alpha subunit homologue (bssA) gene, partial cds	JX219323
Bacterium enrichment culture clone residual oil-degrading OTU1 alkylsuccinate synthase alpha subunit gene, partial cds	KU094062	Uncultured bacterium clone M-CON001 benzylsuccinate synthase alpha subunit (bssA) gene, partial cds	KC463949.1
Bacterium enrichment culture clone octadecane-degrading OTU1 alkylsuccinate synthase alpha subunit gene, partial cds	KU094063	Uncultured bacterium clone M-OIL003 benzylsuccinate synthase alpha subunit (bssA) gene, partial cds	KC464029

Desulfatibacillum aliphaticivorans partial masD gene for methylalkyl succinate synthase, strain CV2803	LN868321.1	*Desulfosporosinus* sp. enrichment culture clone S2S-F11 1.697-4 benzylsuccinate synthase (bssA) gene, partial cds	KJ398020
Desulfatibacillum alkenivorans partial masD gene for methylalkyl succinate synthase, strain PF2803	LN868322	Uncultured bacterium clone OTU1 benzylsuccinate synthase alpha subunit (bssA) gene, partial cds	KX148522.1
Uncultured *Desulfatibacillum* sp. partial masD gene for 1-methyl alkyl succinate synthase, strain Propane60-GuB	LN879422		
Uncultured bacterium partial masD gene for 1-methylalkyl succinate synthase, clone OTU_1	LT546441		
uncultured bacterium partial assA gene for alkylsuccinate synthase, clone HeM4	LT907865.1		

Table S3. Designed Illumina MiSeq adapter primers for *assA* and *bssA* qPCR primers. Adapter sequences are attached to the 5′ end. "MS" denotes MiSeq.

Name	Sequence (**Nextera Adapter Sequence in bold**)	Length (bp)
	bssA forward	
MSbssSuf	**TCGTCGGCAGCGTCAGATGTGTATAAGAGACAG**GAATACGTGGAGCGACCCGCTC	55
MSbssWin	**TCGTCGGCAGCGTCAGATGTGTATAAGAGACAG**CAATCCGTGGCTTCAGGTTCAT	55
MSbssMys	**TCGTCGGCAGCGTCAGATGTGTATAAGAGACAG**CAATCCGTGGCACAACTGCATG	55
MSbssOil	**TCGTCGGCAGCGTCAGATGTGTATAAGAGACAG**GAATCCCTGGTTACAGGTCCAC	55
	bssA reverse	
MSbssHitr	**GTCTCGTGGGCTCGGAGATGTGTATAAGAGACAG**TCCTCGTAGCCTTCCCAGTT	54
	assA forward	
MSassOri	**TCGTCGGCAGCGTCAGATGTGTATAAGAGACAG**CTCCGCCACGGCCAACTG	51
MSassMsd	**TCGTCGGCAGCGTCAGATGTGTATAAGAGACAG**CTCAGCCACCGCCAACTG	51
MSasSml	**TCGTCGGCAGCGTCAGATGTGTATAAGAGACAG**TAGCGCCACGGCCAACTG	51
MSassEx	**TCGTCGGCAGCGTCAGATGTGTATAAGAGACAG**CTCTGCGACCGCGAATTG	51
	assA reverse	
MS8543r	**GTCTCGTGGGCTCGGAGATGTGTATAAGAGACAG**TCGTCRTTGCCCCAYTTNGG	54

Table S4. Examples of sequenced *assA* assay products that passed quality control analysis but that were not annotated as alkylsuccinate synthase genes using a BLASTn analysis.

Organism	Coding DNA Sequence (CDS)	% identity
Polaromonas sp. JS666	SSU ribosomal protein S17P	83
Acidovorax sp. KKS102	Nitrate reductase alpha subunit	87
Rhizobium sp. TCK	RTCK_02902	80
Geobacter sp. M21	Exinuclease ABC; ABC transporter related	78
Sphingobium sp. SCG-1	Aromatic hydrocarbon degradation protein	97
Pseudomonas veronii strain PVy	Bifunctional UDP-4-amino-4-deoxy-L-Arabinose/formyltransferase/UDP-glucuronic acid oxidase ArnA	99
Acidovorax cattleyae strain CAT98_1	Transketolase	84
Rhodoferax sp. DCY110	Transketolase	84
Gemmatimonas aurantiaca T-27	Replicative DNA Helicase	71
Mycobacterium gallinarum JCM 6399	Hypothetical protein	99
Pseudomonas brenneri strain BS2771	UDP-4-amino-4-deoxy-L-Arabinose/formyltransferase / UDP-glucuronic acid dehydrogenase/(UDP-4-keto-hexauronic acid decarboxylating)	99
Mesorhizhobium	ABC transporter	85
Jatrophihabitans sp. GAS493	Dyp-type peroxidase family	81
Rhodoferax saidenbachensis strain DSM 22694	Methionine aminotransferase	82
Sphingomonas sp KC8	Rod-shape-determining protein MreD	73
Rubrivivax gelatinosus strain 1	CtpA cation transport ATPase	76
Geobacter sp. FeAm09	Glutamate synthase large subunit	79
Pandorea pnomenusa strain M202	50S ribosomal protein	81
Paracoccus anguinis strain OM2164	Heavy metal associated domain containing protein	75
Planctomycetes bacterium Pla85_3_4	Hypothetical protein	73
Collimonas fungivorans Ter331	Phosphoribosylformylglycinamidine synthase	85
Noviherbaspirillum sp. UKPF54	Acyl-CoA synthetase	83
Pelolinea submarina MO-CFX1	Simple sugar transport system ATP binding protein	75
Janthinobacterium sp 17J80-10	ABC transporter	73
Sulfuritalea hydrogenivorans sk43H	Hypothetical protein	72
Iamiaceae bacterium SCSIO 58843	Pyruvate dehydrogenase (acetyl-transferring)	80
Jatrophihabitans sp. GAS493	Dyp-type peroxidase family	81
Massilia violaceinigra strain B2	Urocanate hydratase	84
Aquabacterium olei strain NBRC 110486	Acyl transferase	77
Azoarcus sp. DN11	Aspartate ammonia lyase	86

References

[1] Sievers, F.; Wilm, A.; Dineen, D.; Gobson, T.J.; Karplus, K.; Li, W.; Lopez, R.; McWilliam, H.; Remmert, M.; Soding, J.; Thompson, J.D.; Higgins, D.G. Fast, scalable generation of high-quality protein multiple sequence alignments using Clustal Omega. *Mol. Syst. Biol.* **2011**, *7*, 539.

[2] Benchling [Biology Software]. *Retrieved from https://benchling.com* 300, 2017.

[3] Callaghan, A.V.; Davidova, I.A.; Savage-Ashlock, K.; Parisi, V.A.; Gieg, L.M.; Suflita, J.M.; Kukor, J.J.; Wawrik, B. Diversity of benyzl- and alkylsuccinate synthase genes in hydrocarbon-impacted environments and enrichment cultures. *Environ. Sci. Technol.* **2010**, *44*, 7287–7294.

[4] von Netzer, F.; Pilloni, G.; Kleindienst, S.; Krüger, M.; Knittel, K.; Gründger, F.; Lueders, T. Enhanced gene detection assays for fumarate-adding enzymes allow uncovering of anaerobic hydrocarbon degraders in terrestrial and marine systems. *Appl. Environ. Microbiol.* **2013**, *79*, 543–552.

[5] Winderl, C.; Schaefer, S.; Lueders, T. Detection of anaerobic toluene and hydrocarbon degraders in contaminated aquifers using benzylsuccinate synthase (*bssA*) genes as a functional marker. *Environ. Microbiol.* **2007**, *9*, 1035–1046.

[6] Alberta Environment and Parks (AEP). *Alberta Tier 1 Soil and Groundwater Remediation Guidelines*; Land Policy Branch, Policy and Planning Division: Edmonton, AB, Canada, **2019**.
